# Determinants of perceived stress among healthcare workers

**DOI:** 10.1192/j.eurpsy.2026.11815

**Published:** 2026-07-31

**Authors:** I. Sellami, A. Abbes, A. Feki, M. L. Masmoudi, M. Hajjaji, K. Jmal Hammami

**Affiliations:** 1 occupational medicine; 2 Rheumatoloy, Hedi Chaker Hospital, sfax, Tunisia

## Abstract

**Introduction:**

Healthcare workers (HCWs) are frequently exposed to demanding work conditions that can lead to high levels of perceived stress. This stress may compromise their well-being, reduce job satisfaction, and negatively impact the quality of patient care.

**Objectives:**

This study aimed to identify the determinants of perceived stress among HCWs.

**Methods:**

We conducted a descriptive, analytical, cross-sectional survey among HCWs using a self-administered questionnaire. Socio-professional characteristics were collected, job satisfaction was assessed with a single-item measure, and perceived stress was evaluated using the Perceived Stress Scale.

**Results:**

A total of 57 healthcare workers participated in the study with 52.6% of them were female. The mean age of participants was 36.1 ±10.2 years. The mean of the tenure of job was 15 ±11.5 years. The mean of job satisfaction was 3.4 ±1.2. The mean of perceived stress was 19.2 ±11.5. We found 3.5% of participants presented low stress, 93% moderate stress and 3.5% presented high perceived stress. Higher job satisfaction was a protective factor against stress (p = 0.04, r = -0.2).

**Conclusions:**

Job satisfaction had a significant impact on perceived stress among HCWs. Improving work environments is therefore essential to safeguard staff health and enhance the quality of patient care.

**Disclosure of Interest:**

None Declared

